# Systematic Review and Meta-Analysis of Therapeutic Hypothermia in Animal Models of Spinal Cord Injury

**DOI:** 10.1371/journal.pone.0071317

**Published:** 2013-08-09

**Authors:** Peter E. Batchelor, Peta Skeers, Ana Antonic, Taryn E. Wills, David W. Howells, Malcolm R. Macleod, Emily S. Sena

**Affiliations:** 1 Florey Institute of Neuroscience and Mental Health, Heidelberg, Victoria, Australia; 2 Department of Medicine, University of Melbourne, Heidelberg, Victoria, Australia; 3 Division of Clinical Neurosciences, University of Edinburgh, Edinburgh, United Kingdom; University of Pittsburgh, United States of America

## Abstract

**Background:**

Therapeutic hypothermia is a clinically useful neuroprotective therapy for cardiac arrest and neonatal hypoxic ischemic encephalopathy and may potentially be useful for the treatment of other neurological conditions including traumatic spinal cord injury (SCI). The pre-clinical studies evaluating the effectiveness of hypothermia in acute SCI broadly utilise either systemic hypothermia or cooling regional to the site of injury. The literature has not been uniformly positive with conflicting studies of varying quality, some performed decades previously.

**Methods:**

In this study, we systematically review and meta-analyse the literature to determine the efficacy of systemic and regional hypothermia in traumatic SCI, the experimental conditions influencing this efficacy, and the influence of study quality on outcome. Three databases were utilised; PubMed, ISI Web of Science and Embase. Our inclusion criteria consisted of the (i) reporting of efficacy of hypothermia on functional outcome (ii) number of animals and (iii) mean outcome and variance in each group.

**Results:**

Systemic hypothermia improved behavioural outcomes by 24.5% (95% CI 10.2 to 38.8) and a similar magnitude of improvement was seen across a number of high quality studies. The overall behavioural improvement with regional hypothermia was 26.2%, but the variance was wide (95% CI −3.77 to 56.2). This result may reflect a preponderance of positive low quality data, although a preferential effect of hypothermia in ischaemic models of injury may explain some of the disparate data. Sufficient heterogeneity was present between studies of regional hypothermia to reveal a number of factors potentially influencing efficacy, including depth and duration of hypothermia, animal species, and neurobehavioural assessment. However, these factors could reflect the influence of earlier lower quality literature.

**Conclusion:**

Systemic hypothermia appears to be a promising potential method of treating acute SCI on the basis of meta-analysis of the pre-clinical literature and the results of high quality animal studies.

## Introduction

Therapeutic hypothermia has emerged as a clinically useful neuroprotective treatment, substantially improving neurological outcomes following both cardiac arrest and neonatal hypoxic ischaemic encephalopathy. Deep hypothermia has also been shown to prevent catastrophic brain and spinal cord ischaemic injury following induced circulatory arrest [1]. Because of its broad neuroprotective properties, the use of therapeutic hypothermia to treat other neurological conditions has been proposed, including stroke, traumatic brain injury and spinal cord injury (SCI) [1].

In experimental models of traumatic SCI hypothermia is administered either systemically or local to the injury site (regional or epidural hypothermia). The pre-clinical evidence of the effectiveness of hypothermia is somewhat mixed. Some animal studies of systemic hypothermia have reported effectiveness and therapeutic systemic hypothermia has progressed to clinical evaluation where safety has been demonstrated and a trial evaluating its efficacy in cervical SCI is proposed [2], [3], [4]. Similarly, the literature on pre-clinical regional hypothermia contains a variety of studies of varying quality and inconsistent results. Although case reports and small series from some time ago also suggest a potential clinical benefit, a substantial clinical trial utilising regional hypothermia has not been conducted [5], [6].

In other domains, systematic review and meta-analysis have been used to identify the experimental conditions providing maximum efficacy and identify where bias has led to overestimations of treatment effects [7], [8]. Given the conflicting nature of the pre-clinical studies on hypothermia and SCI, we sought to use systematic review and meta-analysis to determine the overall efficacy of systemic and regional hypothermia as well as the impact of varying experimental and quality factors on outcome.

## Methods

### Systematic Review

In November 2011, we searched three electronic databases (Pubmed, EMBASE and ISI Web of Science) using the following search strategy: (hypothermia OR temperature) AND (spinal cord injury OR hemisection OR contusion injury OR dorsal column injury OR complete transection OR corticospinal tract injury). Search results were limited to animals.

### Criteria for Inclusion and Exclusion

We sought controlled studies reporting the efficacy of cooling or hypothermia in *in vivo* animal models of traumatic SCI where outcome was expressed as a change in functional outcome. To be included studies had to report or allow the determination of the number of animals in each group, the mean effect size and its variance. Studies were specifically excluded where non-traumatic models of spinal cord injury were used.

### Data Extraction

From each experiment we extracted data for reported outcomes (functional outcome, lesion volume and preserved tissue). Where a publication reported more than one experiment (for instance the impact of different delays to treatment) we considered these to be independent experiments and extracted data for each of these (correcting the weighting of these studies in meta-analysis to reflect the number of experimental groups served by each control group). Each individual experiment of control cohort versus treatment cohort is herein defined as a comparison. Where different functional outcomes were reported in a single cohort of animals, we combined (nested) these using fixed effects meta-analysis to give a summary estimate of functional outcome in that comparison. However, where different intensities of the same test were carried out in the same cohort of animals, data for the median intensity were used. For sensory tests, only data caudal to the site of the lesion were extracted. Where functional outcome was measured serially we extracted data for the last time point reported.

We assessed study quality according to a 9-point checklist adapted from the consensus statement ‘Good laboratory practice’ in the modelling of stroke and the CAMARADES quality checklist which encompasses the reporting of measures to reduce bias: (i) publication in a peer reviewed journal; (ii) statements describing control of temperature; (iii) randomisation to treatment group; (iv) allocation concealment; (v) blinded assessment of outcome; (vi) avoidance of anaesthetics with known marked intrinsic neuroprotective properties; (vii) sample size calculation; (viii) compliance with animal welfare regulations; (ix) and whether the authors declared any potential conflict of interest. One point was allocated for each of these items.

### Analysis

For each comparison we calculated a normalised effect size as the percentage improvement in outcome in the treatment cohort relative to the control cohort. We then used DerSimonian and Laird random effects weighted mean difference meta-analysis to calculate overall effect size. Results are presented as the summary percentage improvement in outcome in the treatment group compared with the control group, and its 95% confidence intervals. We sought evidence of publication bias using a funnel plot, Egger regression and Trim and Fill [9].

We performed pre-specified stratified meta-analysis to explore the impact on study characteristics on the reported effects of hypothermia. The significance of differences between groups was assessed by partitioning heterogeneity and by using the χ^2^ distribution with n−1 degrees of freedom (df). We explored the impact of the reporting of measures to minimise bias on the complete dataset. We then divided the data into studies reporting regional and those reporting systemic hypothermia. Analysis of these two datasets were performed according to: (i) aspects specific to the modelling of SCI (anaesthesia, species of animal, model and severity of spinal cord injury) (ii) the therapeutic paradigm (depth and duration of hypothermia, time of initiation of hypothermia following injury) and (iii) outcome specific parameters (outcome measure, time of assessment). To account for multiple comparison, we performed a Bonferroni correction and the significance level reduced accordingly from p<0.05.

The protocol for this review, entitled ‘Systematic review and meta-analysis of hypothermia treatment in animal models of traumatic spinal cord injury’ is available on the CAMERADES website www.camerades.info/index_files/Protocols.html.

## Results

### Study Characteristics

**Figure 1:**
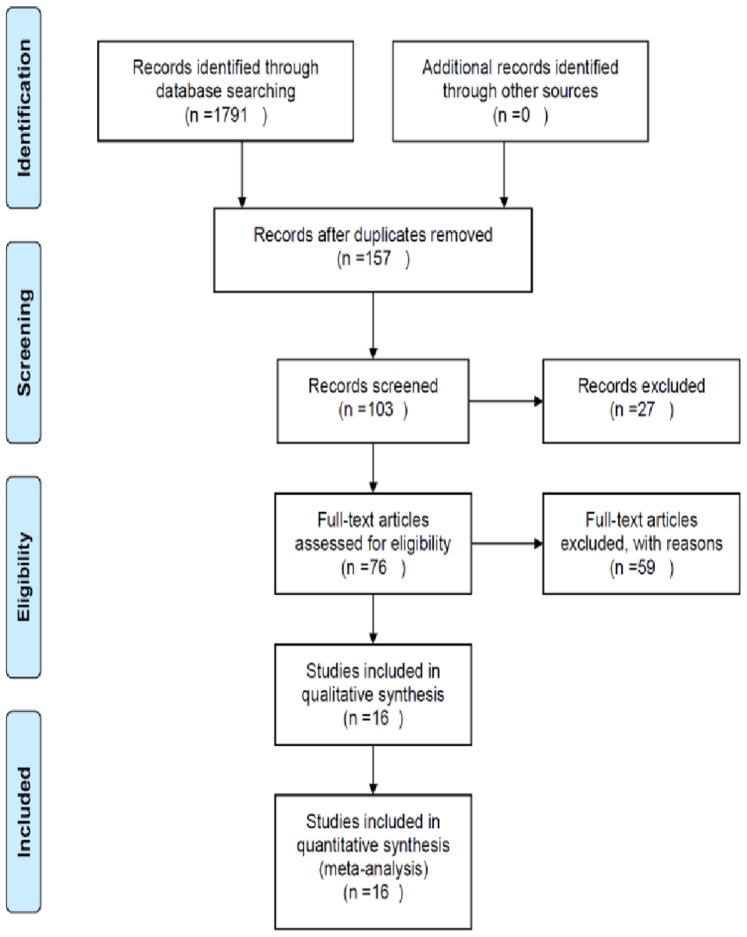
Search strategy outline. Flowchart demonstrating the search strategy used to identify articles for systematic review and meta-analysis of therapeutic hypothermia in animal spinal cord injury models.

Our systematic search identified 1791 publications with 157 potentially relevant articles screened for inclusion. After the removal of duplicate publications and studies deemed not relevant, 76 full text publications were retrieved. Of these 76 publications, 16 met our pre-specified inclusion criteria (Figure 1).

Eight publications explored the use of systemic hypothermia (Martinez-Arizala et al. 1995, Jou et al. 2000, Yu et al. 2000, Westergren et al. 2000, Morino et al. 2008, Lo et al. 2009, Batchelor et al. 2010 and Topuz et al. 2010) [10], [11], [12], [13], [14], [15], [16], [17]. Within these, 14 experiments reported outcome as a change in neurobehavioural score using 241 animals, six experiments reported lesion volume using 71 animals and four experiments reported the degree of preserved tissue in 90 animals. The remaining eight publications used regional hypothermia and these contained 11 separate experiments reporting a change in neurobehavioural score in 207 animals, 3 experiments reported lesion volume in 56 animals, and one experiment reported the degree of preserved tissue in 20 animals (Albin et al. 1967, Albin et al. 1968, Tator & Deecke 1972, Hansebout et al. 1975, Dimar et al. 2000, Casas et al. 2005, Ha et al. 2008 and Morochovic et al. 2008) [18], [19], [20], [21], [22], [23], [24], [25].

**Figure 2:**
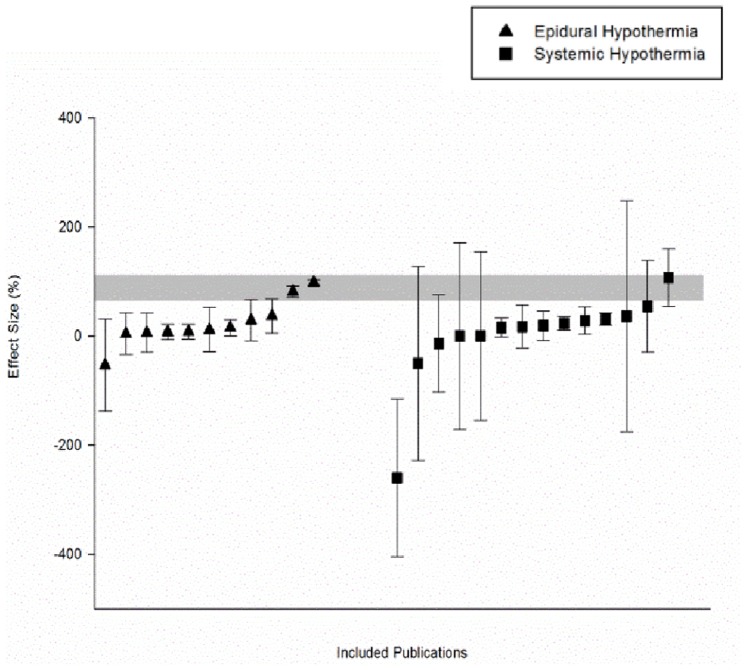
Effect sizes of included publications. Scatter plot demonstrating the distribution of effect sizes (% improvement in neurobiological score) within the included publications (n = 16). Individual experiments that employed a regional cooling approach (n = 11) vs. a systemic cooling approach (n = 14) have been identified and plotted as separate subgroups. The shaded gray bar represents the 95% confidence limits of the global estimate. The vertical error bars represent the 95% confidence intervals for the individual estimates. The width of each bar reflects the log of the number of animals contributing to that comparison.

Overall, therapeutic hypothermia improved neurobehavioral outcomes by 22.8% [95% confidence interval [CI] 2.2 to 43.4] and we observed significant between study heterogeneity (χ^2^ = 562.4, df = 24, p<0.001). Systemic hypothermia was associated with a more precise estimate of effect with significantly less heterogeneity compared to regional hypothermia (systemic: 24.5% [10.2 to 38.8], χ^2^ = 29.1 versus regional: 26.2% [−3.77 to 56.2], χ^2^ = 385, Figure 2).

### Risk of Bias and Publication Bias

**Figure 3:**
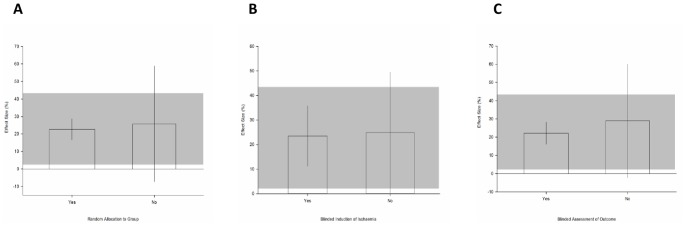
Risk of bias within included studies. Effect of (**A**) random allocation to treatment groups, (**B**) blinded induction of ischaemia and, (**C**) blinded assessment of outcome on improvement in neurobiological score. The shaded gray bar represents the 95% confidence limits of the global estimate. The vertical error bars represent the 95% confidence intervals for the individual estimates. The width of each bar reflects the log of the number of animals contributing to that comparison.

As with animal studies of other neurological disorders, measures to avoid both internal (confounding variables) and external (lack of generalisability) bias are likely to impact on outcome and the utility of these data. We assessed the prevalence of the reporting of measures to reduce bias in the 16 publications reporting neurobehavioural outcome. The median quality score was 4 (inter-quartile range 2–5) out of a possible 9. Studies examining systemic hypothermia had a median quality score of 5 (inter-quartile range 4–6), while studies looking at regional hypothermia had a median quality score of 2 (inter-quartile range 2–4). Eighty-eight percent of the studies were published in peer reviewed journals (14/16). Only one publication reported performing a sample size calculation and this same study was also the only publication to declare a statement of potential conflict of interest, of which there was none. Fifty percent of the publications reported random allocation to treatment group. We observed statistically smaller estimates of effect in these randomised studies (22.6% [16.6 to 28.3], n = 254 animals) compared to non-randomised studies (25.8% [−7.2 to 58.9], n = 193; χ^2^ = 232.5, df = 1, p<10^−51^; Figure 3a). Three publications (19%) reported allocation concealment and in these we also observed smaller estimates of effect (23.5% [11.2 to 35.8], n = 110) compared to those that did not report concealing treatment allocation (24.9% [−0.3 to 49.5], n = 335; χ^2^ = 80.1, df = 1, p<10^−18^; Figure 3b). Forty-four percent (7/16) of the publications reported blinding treatment allocation in the assessment of outcome. Similar to the other parameters used to reduce bias, blinded studies also reported smaller estimates of treatment effects compared to those that did not blind the assessment of outcome (22.2% [16.1 to 28.3], n = 229 animals versus 30.0% [−2.2 to 60.1], n = 219; χ^2^ = 229.1, df = 1, p<10^−33^; Figure 3c).

**Figure 4:**
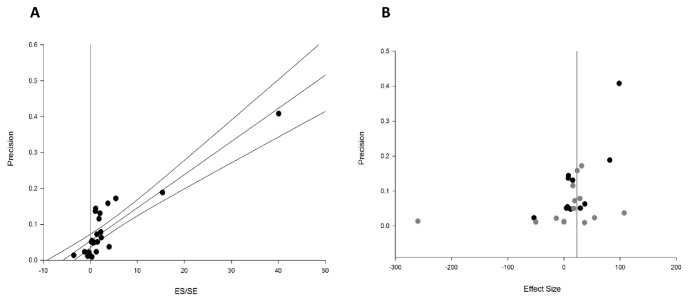
Publication bias. (**A**) Eggar regression showing publication bias within the included studies. (**B**) Funnel plot showing the regional data in black and the systemic data in grey. No additional studies were suggested by trim and fill analysis.

Publication bias was assessed using Eggar regression, together with a funnel plot and trim and fill analysis (Figure 4a, b). A small degree of potential publication bias was detected, suggesting an overstatement of efficacy of 3.3%.

### Systemic Hypothermia

To investigate the limits to the efficacy of systemic hypothermia we assessed the impact of experimental design characteristics on neurobehavioural outcome. In these data, SCI was induced in three experiments using the impactor model, a further three used an impactor plus an additional spacer and eight produced a compression model of SCI with an aneurysm clip. Two experiments reported decompression of the spinal cord by removal of the spacer. One experiment did not report the anaesthetic agent used and the remaining 13 experiments used five different anaesthetic strategies during surgical procedures (halothane, isoflurane, sodium pentothal, ketamine/xylazine, and fluanison/fentanyl). We did not observe a significant effect of these model specific parameters on outcome. We also investigated therapeutic specific parameters. The dose of hypothermia ranged from 28°C to 34°C. Treatment was initiated from 82 minutes before the induction of injury to 30 minutes post injury and the duration of hypothermia ranged from 31 minutes to 7.5 hours. These parameters also did not account for the heterogeneity of outcome. Outcome was assessed between three and 56 days after injury and again no significant influence of times of assessment was observed.

**Figure 5:**
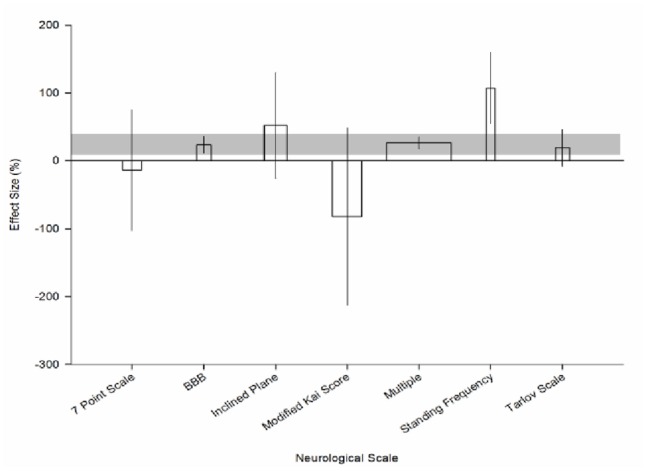
Heterogeneity within systemic hypothermia experiments. Effect of neurobehavioural scale used on effect size reported for experiments employing systemic hypothermia. The shaded gray bar represents the 95% confidence limits of the global estimate. The vertical error bars represent the 95% confidence intervals for the individual estimates. The width of each bar reflects the log of the number of animals contributing to that comparison. Each stratification accounts for a significant proportion of the heterogeneity observed between studies.

The neurobehavioural scale used to assess outcome was the only experimental variable to account for a significant proportion of heterogeneity within the systemic hypothermia dataset (χ^2^ = 18.8, df = 6, p<0.006; Figure 5). We observed the biggest improvement in neurobehavioural outcome where the standing frequency test (107.3% [54.7 to 159.7], n = 12 animals) and inclined plane test (52.0% [−26.1 to 130.2], n = 32) were used. Over a third of animals were assessed using multiple different scales and this strata provided the most precise estimate of effect (26.5% [17.8–35.1], n = 90) followed by the BBB test (23.5% [11.2 to 35.9], n = 20).

### Regional Hypothermia

In the regional hypothermia dataset we also assessed the impact of model specific parameters, treatment specific parameters and outcome specific parameters on neurobehavioural outcome, and identified that all variables assessed accounted for a significant proportion of between study heterogeneity.

#### Model specific parameters

**Figure 6:**
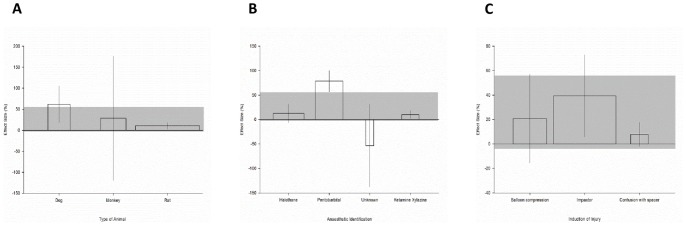
Model specific parameters of regional hypothermia experiments. Effect of (**A**) animal species, (**B**) anaesthetic, and (**C**) induction of injury used, on the effect size observed within experiments utilising regional hypothermia. The shaded gray bar represents the 95% confidence limits of the global estimate. The vertical error bars represent the 95% confidence intervals for the individual estimates. The width of each bar reflects the log of the number of animals contributing to that comparison. Each stratification accounts for a significant proportion of the heterogeneity observed between studies.

Spinal cord injury was modelled in three different species (χ^2^ = 364.3, df = 2, p<0.006, Figure 6a). Greater benefit was observed in dogs (61.9% [18.6 to 105.1], n = 41) and monkeys (28.5% [−119.5 to 176.6], n = 47). The most commonly used species was rats (10.6% [3 to 18.1], n = 119). During SCI modelling, one experiment did not report the anaesthetic agent used and it reported no effect of hypothermia (−53.3% [−137.7–31.1], n = 20; χ^2^ = 362.5, df = 3, p<0.006, Figure 6b). Of the three other strategies employed, the largest improvement in behavioural outcome was observed in studies using pentobarbital (78% [56.9 to 100.0], n = 68), followed by halothane (12.6%, [−6.47 to 31.8], n = 76) and then ketamine/xylazine (10.2% [1.92 to 18.4], n = 43).

Three methods to induce SCI were reported (χ^2^ = 222.1, df = 2, p<0.006, Figure 6c). We observed a 39.4% [5.75 to 73.0) improvement in behavioural outcome in experiments that produced a contusion injury with an impactor (n = 114). We observed smaller effects in both compression injuries induced with balloon inflation (20.8% [−15.4 to 57], n = 61) and where contusion injury was exacerbated by the insertion of a spacer (7.8% [−2.1 to 17.7], n = 32).

#### Treatment specific parameters

**Figure 7:**
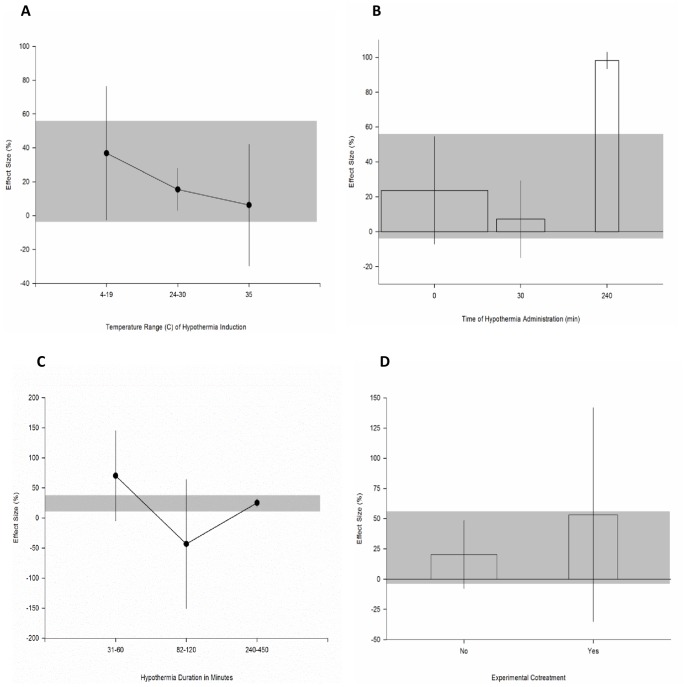
Treatment specific parameters of regional hypothermia experiments. Effect of (**A**) temperature range of hypothermia induction, (**B**) time of hypothermia administration, (**C**) duration of hypothermia and, (**D**) experimental cotreatment on the effect size reported within experiments employing regional hypothermia. The shaded gray bar represents the 95% confidence limits of the global estimate. The vertical error bars represent the 95% confidence intervals for the individual estimates. The width of each bar reflects the log of the number of animals contributing to that comparison. Each stratification accounts for a significant proportion of the heterogeneity observed between studies.

The degree of regional hypothermia delivered ranged from 4°C to 35°C and we observed a clear dose response relationship (χ^2^ = 106.8, df = 2, p<0.006, Figure 7a). Within the temperature range of 4°C to 19°C an improvement of 36.9% ([−2.6 to 76.4], n = 120) was observed. The next strata included animals treated between 24°C and 30°C and we observed an improvement of 15.5% ([3 to 28], n = 96) and the remaining 18 animals were cooled to 35°C where we observed a small improvement (6.2% [−29.7 to 42.1]). Hypothermia was initiated either immediately after the induction of injury, 30 minutes after or 4 hours after injury (χ^2^ = 268.8, df = 2, p<0.006, Figure 7b). The majority of studies initiated treatment at time zero (23.7% [−7 to 54.4], n = 124). The smallest effect was observed at the 30 minute time point (7.2% [−14.8 to 29.3], n = 56) and the largest effect was seen in the single study that initiated treatment at the four hour time point (98.2% [93.4 to 103], n = 27). The duration of cooling ranged from one hour to 120 hours (5 days) (χ^2^ = 304.3, df = 2, p<0.006, Figure 7c). The smallest benefit was observed in the group of animals treated for two hours or less (9.1% [−0.4 to 18.7], n = 52). The largest benefit was observed in the animals treated for up to 3 hours (38.7% [9.3 to 68.2], n = 123) and moderate benefit observed for those treated in the 10 hours to 5 day window (21.9% [2.7 to 41], n = 32).

Only two experiments reported the use of a surgical co-treatment (durotomy and decompression respectively) and this was associated with increased benefit compared to those that used hypothermia treatment alone (53.3% [−35.2 to 141.8], n = 43 versus 20.4% [−7.59 to 48.4], n = 164; χ^2^ = 130.4, df = 1, p<0.006, Figure 7d).

#### Outcome specific parameters

**Figure 8:**
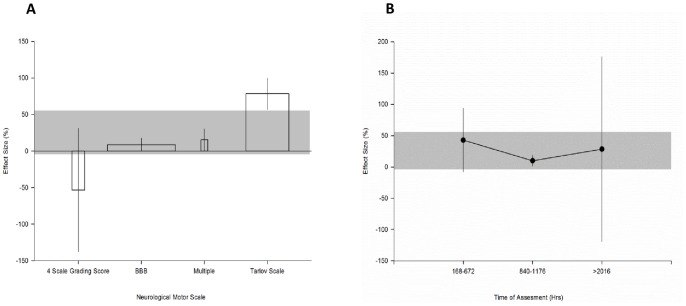
Outcome specific parameters of regional hypothermia experiments. Effect of (**A**) neurobehavioural scale and, (**B**) time of assessment on the reported effect size of experiments employing regional hypothermia. The shaded gray bar represents the 95% confidence limits of the global estimate. The vertical error bars represent the 95% confidence intervals for the individual estimates. The width of each bar reflects the log of the number of animals contributing to that comparison. Each stratification accounts for a significant proportion of the heterogeneity observed between studies.

Four neurobehavioural tests were used to assess outcome (χ^2^ = 363.0, df = 3, p<0.006, Figure 8a). The greatest effect was observed where the Tarlov scale was used (78.3% [56.9 to 100], n = 68). Only one experiment assessed outcome using multiple behavioural tests (15.6% [0.62 to 30.6], n = 69). The most precise estimate of effect was observed with the BBB (8.8% [0.06 to 17.6], n = 108) and no benefit was observed in the single study that used a 4 level grading score (−53%; [137.7 to 31.1], n = 20).

Neurobehavioral scores were assessed seven to 84 days after the induction of SCI (χ^2^ = 317.2, df = 2, p<0.006, Figure 8b). The greatest benefit was observed when outcome was assessed between seven and 28 days after injury (43% [−7.9 to 93.9], n = 51). The majority of animals were assessed in the 35–49 day window (10% [1.3 to 18.6], n = 109) and just two experiments assessed outcome at 84 days (28.5% [−119.5 to 176.6], n = 47).

## Discussion

Using meta-analysis this study examines the use of therapeutic hypothermia in experimental SCI in studies assessing behavioural recovery. Systemic hypothermia improved behavioural outcome by 24.5%, while the improvement with regional hypothermia was 26.2%. Although the data set was relatively small, sufficient heterogeneity (χ^2^ = 385) was present between studies of regional hypothermia to reveal a number of factors potentially influencing the efficacy of hypothermia. In the case of systemic hypothermia, there was limited between study heterogeneity (χ^2^ = 29.1), precluding a broad determination of experimental conditions influencing efficacy.

### Systemic Hypothermia

The benefit of systemic hypothermia in pre-clinical studies demonstrated by meta-analysis supports the favourable view of this therapy noted in a number of recent qualitative systematic reviews [1], [26], [27], [28], [29]. Contrasting with this generally favourable formulation, Kwon et al. (2008) [5], in their systematic review of the field, felt that the pre-clinical literature at the time did not demonstrate a consistent neuroprotective effect with systemic hypothermia. Since the time of this latter review, two further studies with positive outcomes have been published [10], [12]. These studies, together with the study of Yu et al. (2000) [17], have the highest quality (study quality scores of 6/9 or more) of the studies examining systemic hypothermia and SCI, as well as having high external validity. This is reflected in the long survival times of these studies and attempts to mimic human SCI with contusion injuries to the thoracic [17] or cervical cord [12] accompanied by compression in the case of Batchelor et al., 2010 [10]. All three studies demonstrated positive effects on behaviour and tissue preservation and in addition were notable because of their relatively low variance and high statistical precision.

Our search revealed six further (i.e. a total of 9) studies examining behavioural outcomes following systemic hypothermia and SCI. The majority of these remaining studies were of modest internal validity and survival times were generally relatively short. As noted by Kwon, et al. (2008) [5], these studies had conflicting outcomes. It is perhaps worth specifically considering the negative studies. An early study by The Miami Project (published in abstract form) utilising a moderate thoracic injury and level of systemic hypothermia for 4 hrs was negative [13]; however, two subsequent high quality studies by this group using standardised behavioural measures yielded positive results [12], [17]. A study by Westergren et al. (2000) [16] utilising moderate systemic hypothermia for 2 hrs immediately post-injury was also negative. This study had a modest quality score (2/9), very high death rates (50% in the treatment group), utilised a high level of impact injury (arguably limiting the potential for recovery) and had a relatively short survival time of 2 weeks; recovery following severe spinal injury in rats is gradual and plateaus at 6–8 weeks post-injury.

Measures to minimise bias that threaten the internal validity of a study are accepted in the clinical research domain as paramount factors determining study validity [30]. There is evidence that the same is likely to be true in the pre-clinical experiments, with generally smaller effect sizes observed as study quality improves [7], [8], [9], [31], [32], [33], a finding also seen in clinical studies [34]. Poor pre-clinical study quality seems likely to be an important contributor to translational failure and the presence of a number of positive studies examining systemic hypothermia with high quality scores supports moves for translation of this therapy. Clinical trials employing systemic hypothermia in patients with cervical SCI are proposed and pilot data has demonstrated safety in this patient population [2], [3], [4].

Although the overall limited between study heterogeneity with systemic hypothermia restricted analysis of experimental factors influencing study outcome, we did observe that the type of neurobehavioural scale used seemed to influence outcome. The largest magnitudes of improvement were seen with the standing frequency test and inclined plane tests. The standing frequency test was used in a single study [14] and the result may reflect the idiosyncrasy of this scale, with large gains possible with what would otherwise be smaller gains on more conventional motor tests. The inclined plane test was used in three studies, and the large magnitude of improvement reflects the influence of one strongly positive study [15]; the two remaining studies were negative [13], [16] and this is reflected in the large confidence interval. The behavioural data highlights the importance of standardised motor tests to enable comparisons of results and effect sizes across studies.

### Regional Hypothermia

There was substantial heterogeneity between studies employing regional hypothermia. This facilitated the examination of factors influencing outcome. A dose response relationship was evident with temperature, with those studies utilising severe or profound regional hypothermia having larger effect sizes than studies employing moderate-severe regional hypothermia and the latter studies in turn having larger effect than those with mild hypothermia. Although noteworthy, this result may also reflect that studies employing severe or profound regional hypothermia were performed in the 1960’s and 70′s and had relatively low quality scores; the median quality score in the regional hypothermia cohort was 2/9 compared with 5/9 in the systemic hypothermia group. Low study quality has repeatedly been associated with an over-estimation of effect size [7], [8], [9], [31], [32], [33]. In keeping with this latter interpretation, a well conducted (quality score of 6) head to head comparison of differing severities of regional hypothermia failed to demonstrate a neuroprotective benefit for mild, moderate or severe degrees of regional hypothermia [20].

The greater benefit seen in early and less well conducted studies of regional hypothermia may account for the apparent substantially larger benefit of regional hypothermia in large animals assessed using the Tarlov scale, compared with rats undergoing other behaviour assessments. Large animals in these studies also generally received barbiturate anaesthesia and were allowed to survive for 1–4 weeks post-injury, parameters again associated with larger effect sizes.

An interesting alternative explanation for the disparate nature of the data regarding the effectiveness of regional hypothermia was raised by Dimar et al. (2000) [21], who observed a benefit of regional cooling with compressive spinal cord injury produced by an epidural spacer, but no benefit when compression was combined with cord contusion. These data may indicate regional cooling is neuroprotective when a significant degree of cord ischaemia is present (as opposed to cord contusion), an interpretation favoured by Kwon et al. (2008) [5] in their systematic review of the area. The authors felt this theory may explain some of the divergent data and in particular the positive results observed by studies where ischaemia may be a sizable component of injury [21], [23] and the negative results observed by Casas et al. (2003) [35] with a contusion model of injury.

In the current study using meta-regression, larger effect sizes were observed in experiments utilising simple contusion injuries compared with balloon compression where the amount of ischaemic injury would be expected to be greater. This result might argue against hypothermia being neuroprotective with ischaemia. However, examining the individual studies, in the positive study of Hansebout et al. (1975) [23] the authors used balloon compression for 1 h, with hypothermia initiated 15 min post injury (i.e. during the period of compression) and continued for 3.5 h. In two of the negative studies [24], [25], high balloon pressures were used for short periods (5 min) and hypothermia initiated following injury (at 3 h in the case of Tator and Deecke 1972) [25]. Hansebout et al. (1975) [23] used an experimental approach that is similar to the Dimar et al. (2000) study [21], with hypothermia initiated during the period of compression and ischaemia. The positive results of these latter studies support the hypothesis that hypothermia is neuroprotective in the presence of *ongoing* ischaemia and are in keeping with the results of Batchelor et al. (2010) [10] who demonstrated a neuroprotective benefit of *systemic* hypothermia following contusion injury when accompanied by compression, but not following contusion injury alone. These results support the contention that hypothermia is likely to be of most benefit when commenced soon after SCI and continued until compression is relieved, so as to limit additional (and potentially ischaemic) cord injury.

A number of studies utilising regional hypothermia were excluded from analysis. In the majority of cases this was because studies did not have behavioural outcomes or lesion volumes. A few studies were excluded where these factors were examined because no sample variance was presented or could be derived.

### Mechanism of Benefit

Hypothermia is a broad spectrum neuroprotective therapy with multiple mechanisms of action reported in SCI including reductions in haemorrhage [6], oedema [36], pressure [37], possibly glutamate excitotoxicity (Farooque et al., 1997 [38] results contrasting with those of Yamamoto et al., 1998 [39]), oxidative stress and inflammation [40] as well as apoptosis [41]. The heterogeneity of outcomes in these studies evaluating the pathophysiological mechanisms of hypothermia for the most part precluded their inclusion in the current meta-analysis. Exactly which pathophysiological mechanisms are most responsible for neuroprotective effects is unclear, although outcome appears to correlate with the control of local pressure around the injured spinal cord with hypothermia [37].

### Conclusion

Systemic hypothermia appears to be a promising potential method of treating acute SCI on the basis of meta-analysis of the pre-clinical literature and the results of high quality animal studies. Although meta-analysis of the pre-clinical literature on regional hypothermia also suggests benefit, this result may reflect a preponderance of low quality studies performed many years ago, with negative more recent high quality data. A preferential effect of hypothermia in ischaemic models of injury could also explain some but not all of the disparate data on regional hypothermia. The relatively robust data on systemic hypothermia suggest translation potential, although pre-clinical studies have utilised hypothermia relatively soon after injury and for shorter durations than that proposed for future clinical study.
